# Estimating the Moisture Ratio Model of Cantaloupe Slices by Maximum Likelihood Principle-Based Algorithms

**DOI:** 10.3390/plants12040941

**Published:** 2023-02-19

**Authors:** Guanyu Zhu, G. S. V. Raghavan, Zhenfeng Li

**Affiliations:** 1 Jiangsu Key Laboratory of Advanced Food Manufacturing Equipment and Technology, School of Mechanical Engineering, Jiangnan University, Wuxi 214122, China; 2 Department of Bioresource Engineering, McGill University, 21111 Lakeshore Road, Sainte-Anne-de-Bellevue, QC H9X 3V9, Canada

**Keywords:** cantaloupe, moisture ratio model, maximum likelihood principle, image processing, microwave drying system

## Abstract

As an agricultural plant, the cantaloupe contains rich nutrition and high moisture content. In this paper, the estimation problem of the moisture ratio model during a cantaloupe microwave drying process was considered. First of all, an image processing-based cantaloupe drying system was designed and the expression of the moisture ratio with regard to the shrinkage was built. Secondly, a maximum likelihood principle-based iterative evolution (MLP-IE) algorithm was put forward to estimate the moisture ratio model. After that, aiming at enhancing the model fitting ability of the MLP-IE algorithm, a maximum likelihood principle-based improved iterative evolution (MLP-I-IE) algorithm was proposed by designing the improved mutation strategy, the improved scaling factor, and the improved crossover rate. Finally, the MLP-IE algorithm and MLP-I-IE algorithm were applied for estimating the moisture ratio model of cantaloupe slices. The results showed that both the MLP-IE algorithm and MLP-I-IE algorithm were effective and that the MLP-I-IE algorithm performed better than the MLP-IE algorithm in model estimation and validation.

## 1. Introduction

A cantaloupe (*Cucumis melo* var. *saccharinus*) is a kind of agricultural plant and belongs to Cucurbitaceae. Cantaloupes contain rich nutrition and are good sources of ζ-carotene and vitamin C. Furthermore, they have low unsaturated fat, low cholesterol, and high moisture contents [1,2,3,4]. China is the largest producer of cantaloupes with a production of 13.8 million tons in 2020 [5]. The rot of cantaloupes caused by untimely transportation and sales results in losses of up to 20–30% of total production [6]. Drying is a common method to effectively extend the shelf life of cantaloupes. In drying processes, the changes in the moisture contents always lead to changes in shapes which are expressed as shrinkage [7,8]. In the process of modeling drying processes, the moisture content and the shrinkage are two significant indicators [9,10]. In recent decades, the different mathematical models of moisture content and shrinkage during drying processes have been studied [11,12,13]. Yadollahinia et al. investigated the drying characteristics of potato slices and concluded that the dimensionless area changes of the potato slices decreased linearly as the dimensionless moisture content decreased [14]. During the drying process of pineapple slices, a linear function containing exponential constants was used to express the relationship between the shrinkage and the moisture content [15]. The models in [14,15] were the shrinkage versus the moisture content. Conversely, this paper constructed the mathematical model of the moisture ratio with regard to the shrinkage during the cantaloupe drying process as the *n*-order polynomial according to Weierstrass approximation theorem, and developed two new algorithms for model estimation.

The maximum likelihood principle is widely utilized in system modellng and parameter estimation for the reason that it has good statistical properties and it can be applied for both linear models and nonlinear models [16,17]. In order to obtain the estimated values, the maximum likelihood principle is to maximize the probability of the occurrence of the experimental data [18,19,20]. Based on the maximum likelihood principle, Wu et al. studied a method to jointly estimate the amplitude and noise variance of a single sinusoid [21]. Xie et al. used the maximum likelihood principle to reconstruct the parameters of Bernoulli autoregressive systems [22]. Çayır and Candan developed a parameter estimation approach for autoregressive models through using the maximum likelihood principle [23]. By using the maximum likelihood principle, this paper derived the maximum likelihood fitness and proposes two novel algorithms to estimate the moisture ratio model of a cantaloupe drying process.

The steps of population iterative evolution algorithms are similar to biological evolution processes, and the goal of population iterative evolution algorithms is to generate an individual who has the best adaptability to the environment [24,25]. Specifically, the population iterative evolution algorithms are iterative processes in which the individuals evolve as the increase of generation from a randomly selected initial population [26,27]. The differential evolution algorithm is one of the population iterative evolution algorithms. It is easy to realize and performs well in finding the best solutions to complex optimization problems [28,29]. There are three main steps of the differential evolution algorithm, including the mutation process, the crossover process, and the selection process [30]. Recently, by using the differential evolution algorithm and designing a specific mutation strategy, Stanovov et al. developed a dual-population algorithmic scheme [31]. Combining the differential evolution algorithm and the extended Kalman filter, Xiao et al. presented a joint algorithm for the state of charge estimation [32]. This paper took the maximum likelihood fitness in the differential evolution algorithm and thus derived two novel algorithms for model estimation.

The main contributions of this paper are listed as follows.

1. An image processing-based cantaloupe drying system was designed to generate the experimental data and the expression of the moisture ratio with regard to the shrinkage in the drying process of cantaloupe slices was built in line with the Weierstrass approximation theorem.
2. Through deducing the maximum likelihood fitness, a maximum likelihood principle-based iterative evolution (MLP-IE) algorithm was developed to estimate the moisture ratio model.
3. Aiming at enhancing the model fitting ability of the MLP-IE algorithm, a maximum likelihood principle-based improved iterative evolution (MLP-I-IE) algorithm was proposed by designing the improved mutation strategy, the improved scaling factor, and the improved crossover rate.
4. The MLP-IE algorithm and MLP-I-IE algorithm were applied for estimating the moisture ratio model of cantaloupe slices. The results showed that both the two proposed algorithms were effective and that the MLP-I-IE algorithm performed better than the MLP-IE algorithm in model estimation and validation.

## 2. Drying Process of Cantaloupe Slices

### 2.1. Design of Cantaloupe Microwave Drying System Based on Image Processing

In recent years, image processing has been widely used in a variety of drying systems [33,34]. By constructing the expression between the visual appearances such as the color, size, and shape, and the easily measured quality attributes such as the moisture content, density, and porosity at different stages of drying processes, image processing has been used to evaluate the qualities of dried products at a specific time in drying processes [35,36].

The fresh cantaloupes used in the experiments were purchased at a local market in Wuxi, China. Undamaged cantaloupes were selected and stored at 5 °C and then placed at room temperature at 20 °C for 30 min before drying. They were washed, peeled, and cut into 6 mm slices using a mechanical cutter. The slices were then cut into 25 mm diameter cylindrical pieces with a cutting tool. The initial moisture content of fresh cantaloupe samples was measured as 9.87 g/g on the dry basis by drying with hot air at 105 °C for 24 h, which was adequate to obtain the constant mass of the slices.

The developed experiment system consisted of an image processing unit and a microwave drying unit. In the image processing part, 3 LED light strips were applied as light sources, which were installed on the door of the microwave oven. A hole with a diameter of 6 mm was drilled in the top of the microwave oven for imaging, and an industrial camera (SKT-SL1200C-123A, Chengyishun Tech. Co. Ltd., Shenzhen, China) was installed above the hole. The collected images were transmitted to the PC via a USB cable. As for the microwave drying part, a 700 W microwave oven (EM7KCGW3-NR, Midea Co. Ltd., Guangzhou, China) was utilized for drying, where the original circuit was modified to make the microwave power continuously adjustable with the help of a Triac and a data acquisition (DAQ) board (USB 6008, National Instruments Corp., Austin, TX, USA). The sample holding plate was supported by an electric balance above the microwave oven cavity through four Teflon sticks for mass measurement. The electronic scale was able to read the mass information in real time and transfer the data to the PC through the RS232 to USB cable. The precision of the electronic balance was 0.01 g. An optical fiber sensor (HQFTS-PAA0A-0300, Xian Heqi Photoelectric Tech. Co. Ltd., Xi’an, China) was inserted into the core of one of the samples to take temperature [37]. The collected optical signal representing the temperature was converted into a DC voltage signal through the temperature transmitter and was recognized by the PC via the DAQ module.

The drying scheme was microwave drying at a constant temperature of 60 °C. The temperature of the material core was measured in real-time by the optical fiber, and the constant drying temperature of 60 °C was achieved by continuously adjusting the power under a PID control strategy [38]. Mass was measured and the image processing algorithm was performed every 30 s, followed by data recording. Drying was stopped when the dry basis moisture content reached 0.176 g/g, that is, the sample mass detected by the electronic scale reached the corresponding converted mass.

**Figure 1:**
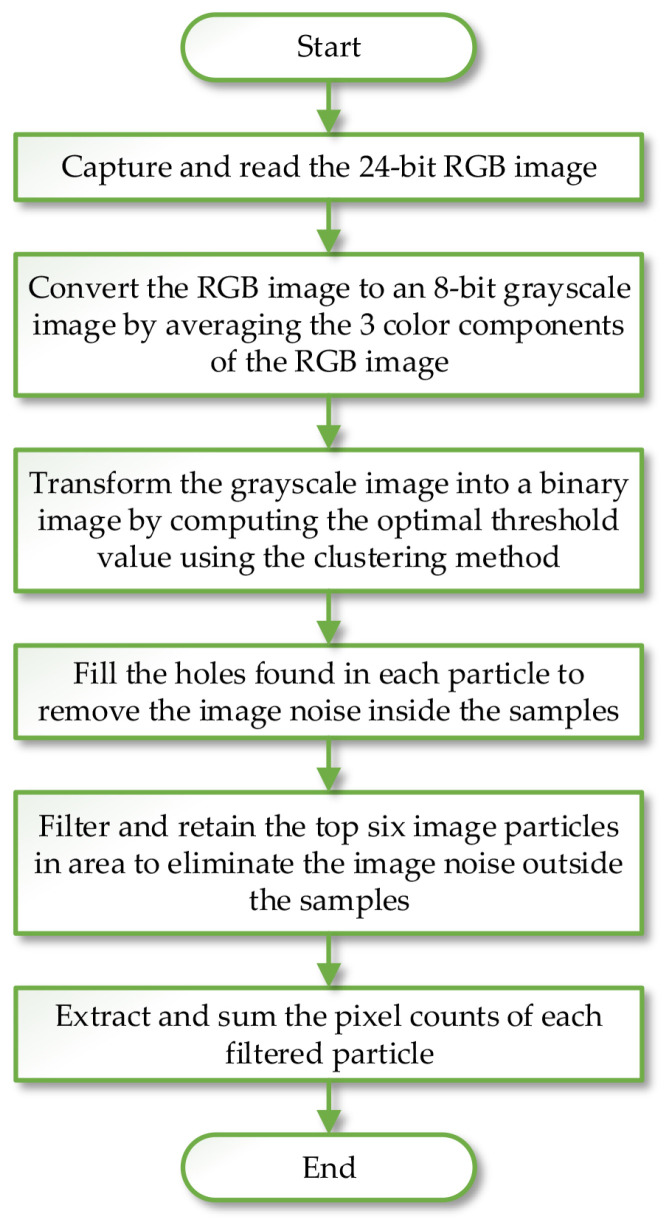
The procedure of the image processing.

**Figure 2:**
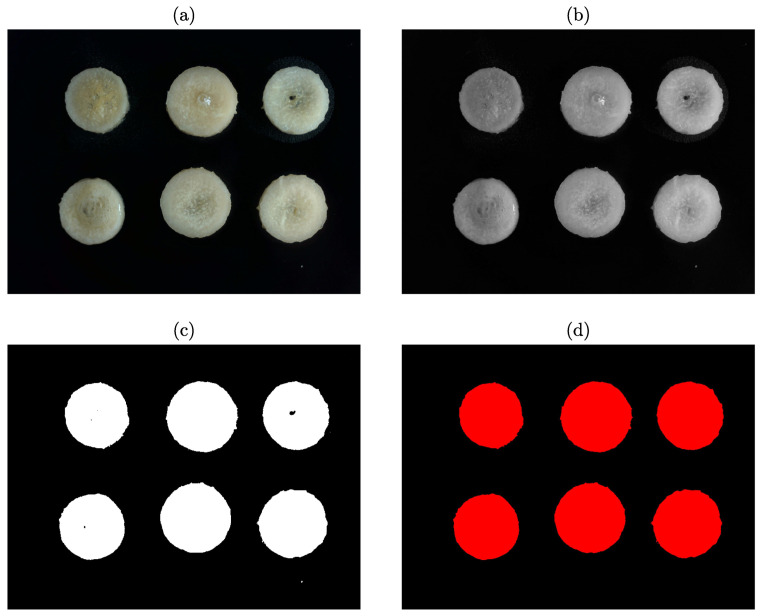
The image-processing steps: (**a**) original RGB image before processing; (**b**) grayscale image; (**c**) binary image; (**d**) image after the operation of filling holes and filtering particles.

An image processing algorithm was developed to monitor the shrinkage of the material. The software, Vision and Motion Module based on LabVIEW (Version 16.0; National Instruments Corp., Austin, TX, USA), was utilized to implement the algorithm. The procedure of the image processing is summarized in Figure 1, and a typical example of the steps is illustrated in Figure 2.

### 2.2. Moisture Ratio Model of Cantaloupe Slices

During the microwave-drying process, the moisture content $M_{C}\left(t\right)$ and the moisture ratio $M_{R}\left(t\right)$ of the cantaloupe samples were defined as

$$M_{C}\left(t\right)=\frac{m\left(t\right)-m_{d}}{m_{d}},\;M_{R}\left(t\right)=\frac{M_{C}\left(t\right)}{M_{C}\left(0\right)},$$

where *t* is the drying time (min), m(t) is the real mass of the samples at time *t* (g), $m_{d}$ is the mass of dry matter of the samples (g), $M_{C}\left(t\right)$ is the moisture content at time *t* (g/g), $M_{C}\left(0\right)$ is the initial moisture content at time t=0 (g/g), and $M_{R}\left(t\right)$ is the moisture ratio at time *t* (dimensionless).

The shrinkage of the samples (i.e., the area ratio) was defined as

$$S\left(t\right)=\frac{A(t)}{A(0)},$$

where A(t) is the area at time *t*, which is expressed by the number of pixels of the samples (px), A(0) is the initial area of the samples (px), and S(t) is the shrinkage of the samples at time *t* (dimensionless).

The moisture ratio $M_{R}\left(t\right)$ could be expressed as a function of the shrinkage S(t):

(1) $$M_{R}\left(t\right)=f\left(S\left(t\right)\right)+w\left(t\right),$$

where $M_{R}\left(t\right)$ is the experimental moisture ratio, S(t) is the shrinkage of the samples, f(S(t)) is the function value, and w(t) is a white Gaussian noise with variance $\sigma^{2}$.

**Theorem** **1.** *(Weierstrass approximation theorem): Let f(x) be continuous on an interval C. Then, for any ϵ>0, there exists a polynomial μ(x) such that*

|μ(x)−f(x)|<ϵ,∀x∈C.

According to Theorem 1, it can be deduced that any continuous function f(x) can be approximated arbitrarily by a polynomial μ(x) with the required accuracy. Compared with other models, such as a linear model, a quadratic polynomial, an exponential model, and so on, the polynomial model can be applied more widely in describing or predicting the moisture ratio models of different materials under different drying conditions and processes. This is because the polynomial model can approximate any type of model as stated in Theorem 1. (Weierstrass approximation theorem). By replacing f(S(t)) in (1) with a *n*-order polynomial μ(S(t)), Equation (1) could be reritten as

(2) $$\begin{array}{ccc} M_{R}\left(t\right) & = & \mu (S(t))+w(t)\\ & = & r_{0}+r_{1}S\left(t\right)+r_{2}S^{2}\left(t\right)+\cdots +r_{n}S^{n}\left(t\right)+w\left(t\right). \end{array}$$

The parameter vector γ and the information vector τ(t) were defined as

$$\begin{array}{ccc} \gamma & = & {\left[r_{0},r_{1},r_{2},\cdots ,r_{n}\right]}^{T}\in R^{Q},\;Q=n+1,\\ \tau (t) & = & {\left[1,S\left(t\right),S^{2}\left(t\right),\cdots ,S^{n}\left(t\right)\right]}^{T}\in R^{Q}. \end{array}$$

Therefore, Equation (2) could be reritten as

(3) $$\begin{array}{ccc} M_{R}\left(t\right) & = & \mu (S(t))+w(t)\\ & = & \tau^{T}\left(t\right)\gamma +w\left(t\right). \end{array}$$

The goal of this paper is to propose two new algorithms to estimate the model in (3) from a batch of discrete experimental data $\{S\left(t_{k}\right)$, $M_{R}\left(t_{k}\right):\;$k=1, 2, ⋯, D}. In addition, in this paper, we utilized the uniform sampling method with the sampling period Δ. Thus the experimental data could be represented as {S(kΔ), $M_{R}\left(k\Delta \right):\;$k=1, 2, ⋯, $k_{\max}\}$ or {S(k), $M_{R}\left(k\right):\;$k=1, 2, ⋯, $k_{\max}\}$ for short.

## 3. MLP-IE Algorithm

This section developed the maximum likelihood principle-based iterative evolution (MLP-IE) algorithm to estimate the parameter vector γ of the model in (3). There are five main stages of the MLP-IE algorithm, including the population initialization, the mutation stage, the crossover stage, the derivation of maximum likelihood fitness, and the selection stage.

### 3.1. Population Initialization

The MLP-IE algorithm started with the generation of an initial population. Considering the *Q*-dimensional parameter vector to be estimated γ in (3), the population size was supposed to be P∈R and the initial population was defined as

(4) $${\hat{\Gamma }}^{0}={\left[{\hat{\gamma }}_{1}^{0},{\hat{\gamma }}_{2}^{0},\cdots ,{\hat{\gamma }}_{P}^{0}\right]}^{T}\in R^{P\times Q},$$

which contains *P* initial individuals (i.e., the parameter vectors to be estimated) from ${\hat{\gamma }}_{1}^{0}$ to ${\hat{\gamma }}_{P}^{0}$. In the initial population ${\hat{\Gamma }}^{0}$, the *p*th initial individual was

(5) $${\hat{\gamma }}_{p}^{0}={\left[{\hat{\gamma }}_{p,1}^{0},{\hat{\gamma }}_{p,2}^{0},\cdots ,{\hat{\gamma }}_{p,Q}^{0}\right]}^{T}\in R^{Q},\;p=1,2,\cdots ,P,$$

where *p* is the index for the individuals. Each element in the *p*th initial individual ${\hat{\gamma }}_{p}^{0}$ was randomly generated as follows:

(6) $${\hat{\gamma }}_{p,q}^{0}=\mathrm{rand}\left(0,1\right),\;q=1,2,\cdots ,Q,$$

where *q* is the index for the elements in the *p*th individual and rand(0,1) is a uniformly distributed stochastic number between 0 and 1. Thus the population initialization was completed.

Because the population and the individuals were changed with the different evolution generations, we defined the population ${\hat{\Gamma }}^{g}$ and the individual ${\hat{\gamma }}_{p}^{g}$ at the generation $g\in [0,g_{\max}-1]$ as

(7) $$\begin{array}{ccc} {\hat{\Gamma }}^{g} & = & {\left[{\hat{\gamma }}_{1}^{g},{\hat{\gamma }}_{2}^{g},\cdots ,{\hat{\gamma }}_{P}^{g}\right]}^{T}, \end{array}$$

(8) $$\begin{array}{ccc} {\hat{\gamma }}_{p}^{g} & = & {\left[{\hat{\gamma }}_{p,1}^{g},{\hat{\gamma }}_{p,2}^{g},\cdots ,{\hat{\gamma }}_{p,Q}^{g}\right]}^{T}. \end{array}$$

Each individual ${\hat{\gamma }}_{p}^{g}$ in the population ${\hat{\Gamma }}^{g}$ is a possible estimate of the parameter vector γ.

### 3.2. Mutation Stage

After the population initialization, the mutation stage was realized to produce the mutant vector for each individual ${\hat{\gamma }}_{p}^{g}$.

The mutant vector ${\hat{\zeta }}_{p}^{g}$ was defined as

(9) $${\hat{\zeta }}_{p}^{g}={\left[{\hat{\zeta }}_{p,1}^{g},{\hat{\zeta }}_{p,2}^{g},\cdots ,{\hat{\zeta }}_{p,Q}^{g}\right]}^{T}\in R^{Q}.$$

At this stage, the mutant vector ${\hat{\zeta }}_{p}^{g+1}$ was produced by adding the vectorial difference between the second and the third individuals to the first individual:

(10) $${\hat{\zeta }}_{p}^{g+1}={\hat{\gamma }}_{\kappa_{1}}^{g}+F\cdot \left({\hat{\gamma }}_{\kappa_{2}}^{g}-{\hat{\gamma }}_{\kappa_{3}}^{g}\right),\;\kappa_{1},\kappa_{2},\kappa_{3}\in \left[1,P\right],$$

where F∈R is a positive constant called the scaling factor which controls the magnitude of the vectorial difference ${\hat{\gamma }}_{\kappa_{2}}^{g}-{\hat{\gamma }}_{\kappa_{3}}^{g}$, and $\kappa_{1}$, $\kappa_{2}$, and $\kappa_{3}$ are integers stochastically selected from the set {1, 2, ⋯, P} and those three integers $\kappa_{1}$, $\kappa_{2}$, and $\kappa_{3}$ are not equal to each other and not equal to the index *p*.

### 3.3. Crossover Stage

After producing the mutant vector ${\hat{\zeta }}_{p}^{g+1}$ during the mutation stage, the crossover stage was implemented to enhance population diversity. The crossover vector ${\hat{\eta }}_{p}^{g}$ was defined as

(11) $${\hat{\eta }}_{p}^{g}={\left[{\hat{\eta }}_{p,1}^{g},{\hat{\eta }}_{p,2}^{g},\cdots ,{\hat{\eta }}_{p,Q}^{g}\right]}^{T}\in R^{Q}.$$

In the crossover stage, some elements in the mutant vector ${\hat{\zeta }}_{p}^{g+1}$ and some elements in the individual ${\hat{\gamma }}_{p}^{g}$ were mixed to construct the crossover vector ${\hat{\eta }}_{p}^{g+1}$. The scheme for generating every element in the crossover vector ${\hat{\eta }}_{p}^{g+1}$ was shown as follows:

(12) $$\begin{array}{ccc} {\hat{\eta }}_{p,q}^{g+1} & = & \left\{\begin{array}{cc} {\hat{\zeta }}_{p,q}^{g+1}, & \mathrm{if}\;\mathrm{rand}\left(0,1\right)<C_{R}\;\mathrm{or}\;q=q_{\mathrm{rand}}\\ {\hat{\gamma }}_{p,q}^{g}, & \mathrm{if}\;\mathrm{rand}\left(0,1\right)⩾C_{R}\;\mathrm{and}\;q\neq q_{\mathrm{rand}} \end{array}\right., \end{array}$$

where $C_{R}\in R$ is a positive constant called the crossover rate which controls the probability of preserving elements in the mutant vector ${\hat{\zeta }}_{p}^{g+1}$ or the individual ${\hat{\gamma }}_{p}^{g}$, and $q_{\mathrm{rand}}$, which is to ensure that the crossover vector ${\hat{\eta }}_{p}^{g+1}$ obtains at least one element in the mutant vector ${\hat{\zeta }}_{p}^{g+1}$, is a integer stochastically selected from the set {1, 2, ⋯, $k_{\max}\}$ and newly produced for each index *p*.

### 3.4. Derivation of Maximum Likelihood Fitness

On the basis of the maximum likelihood principle, the maximum likelihood fitness (MLF) was deduced for the MLP-IE algorithm in this subsection.

For the discrete experimental data {S(k), $M_{R}\left(k\right):\;$k=1, 2, ⋯, $k_{\max}\}$, the likelihood function $L(M_{R}\left(1\right)$, $M_{R}\left(2\right)$, ⋯, $M_{R}\left(k_{\max}\right)|S\left(1\right)$, S(2), ⋯, $S(k_{\max})$, γ) was equal to the joint conditional probability density function of $\{M_{R}\left(1\right)$, $M_{R}\left(2\right)$, ⋯, $M_{R}\left(k_{\max}\right)\}$ with the given {S(1), S(2), ⋯, $S(k_{\max})\}$ and γ:

(13) $$\begin{array}{ccc} & & L(M_{R}\left(1\right),M_{R}\left(2\right),\cdots ,M_{R}\left(k_{\max}\right)|S\left(1\right),S\left(2\right),\cdots ,S\left(k_{\max}\right),\gamma )\\ & = & p(M_{R}\left(1\right),M_{R}\left(2\right),\cdots ,M_{R}\left(k_{\max}\right)|S\left(1\right),S\left(2\right),\cdots ,S\left(k_{\max}\right),\gamma )\\ & = & p(M_{R}\left(k_{\max}\right)|M_{R}\left(1\right),M_{R}\left(2\right),\cdots ,M_{R}\left(k_{\max}-1\right),S\left(1\right),S\left(2\right),\cdots ,S\left(k_{\max}\right),\gamma )\\ & & \times p(M_{R}\left(k_{\max}-1\right)|M_{R}\left(1\right),M_{R}\left(2\right),\cdots ,M_{R}\left(k_{\max}-2\right),S\left(1\right),S\left(2\right),\cdots ,S\left(k_{\max}-1\right),\gamma )\\ & & \times \cdots \times p(M_{R}\left(1\right)|M_{R}\left(0\right),S\left(1\right),\gamma )\\ & = & \prod_{k=1}^{k_{\max}}p\left(\tau^{T}\left(k\right)\gamma +w\left(k\right)|M_{R}\left(1\right),M_{R}\left(2\right),\cdots ,M_{R}\left(k_{\max}-1\right),S\left(1\right),S\left(2\right),\cdots ,S\left(k\right),\gamma \right). \end{array}$$

For the reason that w(k) was white Gaussian noise and was independent of $\{M_{R}\left(1\right)$, $M_{R}\left(2\right)$, ⋯, $M_{R}\left(k_{\max}-1\right)\}$, {S(1), S(2), ⋯, $S(k_{\max})\}$ and γ, Equation (13) could be rewritten as

(14) $$\begin{array}{ccc} & & L(M_{R}\left(1\right),M_{R}\left(2\right),\cdots ,M_{R}\left(k_{\max}\right)|S\left(1\right),S\left(2\right),\cdots ,S\left(k_{\max}\right),\gamma )\\ & = & \prod_{k=1}^{k_{\max}}p\left(w\left(k\right)\right)+h\\ & = & {\left(2\pi \sigma^{2}\right)}^{-\frac{k_{\max}}{2}}\exp\left(-\frac{1}{2\sigma^{2}}\sum_{t=1}^{k_{\max}}w^{2}\left(k\right)\right)+h, \end{array}$$

where *h* denotes a constant. The goal was to maximize the likelihood function $L(M_{R}\left(1\right)$, $M_{R}\left(2\right)$, ⋯, $M_{R}\left(k_{\max}\right)|S\left(1\right)$, S(2), ⋯, $S(k_{\max})$, γ) in (14) to maximize the joint conditional probability density function of $\{M_{R}\left(1\right)$, $M_{R}\left(2\right)$, ⋯, $M_{R}\left(k_{\max}\right)\}$ with the given {S(1), S(2), ⋯, $S(k_{\max})\}$ and γ. Nevertheless, the above operation was difficult to realize due to the huge computational burden. For purpose of tackling this issue, we could take the logarithm of the likelihood function $L(M_{R}\left(1\right)$, $M_{R}\left(2\right)$, ⋯, $M_{R}\left(k_{\max}\right)|S\left(1\right)$, S(2), ⋯, $S(k_{\max})$, γ) in (14) and equivalently maximized the logarithm likelihood function. That logarithm likelihood function was calculated by

(15) $$\begin{array}{ccc} & & l(M_{R}\left(1\right),M_{R}\left(2\right),\cdots ,M_{R}\left(k_{\max}\right)|S\left(1\right),S\left(2\right),\cdots ,S\left(k_{\max}\right),\gamma )\\ & = & \ln L(M_{R}\left(1\right),M_{R}\left(2\right),\cdots ,M_{R}\left(k_{\max}\right)|S\left(1\right),S\left(2\right),\cdots ,S\left(k_{\max}\right),\gamma )\\ & = & -\frac{k_{\max}}{2}\ln\left(2\pi \sigma^{2}\right)-\frac{1}{2\sigma^{2}}\sum_{k=1}^{k_{\max}}w^{2}\left(k\right)+\ln h. \end{array}$$

To maximize the logarithm likelihood function $l(M_{R}\left(1\right)$, $M_{R}\left(2\right)$, ⋯, $M_{R}\left(k_{\max}\right)|S\left(1\right)$, S(2), ⋯, $S(k_{\max})$, γ) in (15), we made its derivative equal zero and obtained the solution

(16) $$\sigma^{2}=\frac{1}{k_{\max}}\sum_{k=1}^{k_{\max}}w^{2}\left(k\right).$$

Inserting (16) into (15) gave

(17) $$\begin{array}{ccc} & & l(M_{R}\left(1\right),M_{R}\left(2\right),\cdots ,M_{R}\left(k_{\max}\right)|S\left(1\right),S\left(2\right),\cdots ,S\left(k_{\max}\right),\gamma )\\ & = & -\frac{k_{\max}}{2}\ln\left(2\pi \right)-\frac{k_{\max}}{2}+\ln h-\frac{k_{\max}}{2}\ln\frac{1}{k_{\max}}\sum_{k=1}^{k_{\max}}w^{2}\left(k\right)\\ & = & \mathrm{const}-\frac{k_{\max}}{2}\ln\frac{1}{k_{\max}}\sum_{k=1}^{k_{\max}}w^{2}\left(k\right). \end{array}$$

In view of (17) and (3), the MLF λ(γ) was defined as

$$\begin{array}{ccc} \lambda (\gamma ) & = & \frac{1}{k_{\max}}\sum_{k=1}^{k_{\max}}{\left[M_{R}\left(k\right)-\tau^{T}\left(k\right)\gamma \right]}^{2}. \end{array}$$

Therefore, the logarithm likelihood function $l(M_{R}\left(1\right)$, $M_{R}\left(2\right)$, ⋯, $M_{R}\left(k_{\max}\right)|S\left(1\right)$, S(2), ⋯, $S(k_{\max})$, γ) in (17) could be rewritten as

(18) $$l\left(M_{R}\left(1\right),M_{R}\left(2\right),\cdots ,M_{R}\left(k_{\max}\right)|S\left(1\right),S\left(2\right),\cdots ,S\left(k_{\max}\right),\gamma \right)=\mathrm{const}-\frac{k_{\max}}{2}\ln\lambda \left(\gamma \right).$$

From (18), it could be observed that the maximum value of the logarithm likelihood function $l(M_{R}\left(1\right)$, $M_{R}\left(2\right)$, ⋯, $M_{R}\left(k_{\max}\right)|S\left(1\right)$, S(2), ⋯, $S(k_{\max})$, γ) could be obtained through minimizing the MLF,

(19) $$\lambda \left(\gamma \right)=\frac{1}{k_{\max}}\sum_{k=1}^{k_{\max}}{\left[M_{R}\left(k\right)-\tau^{T}\left(k\right)\gamma \right]}^{2}=\min.$$

### 3.5. Selection Stage

At this stage, the fitness of the crossover vector ${\hat{\eta }}_{p}^{g+1}$ and the individual ${\hat{\gamma }}_{p}^{g}$ was assessed by calculating their MLFs $\lambda ({\hat{\eta }}_{p}^{g+1})$ and $\lambda ({\hat{\gamma }}_{p}^{g})$ and the greedy selection strategy was adopted to determine whether the crossover vector ${\hat{\eta }}_{p}^{g+1}$ or the individual ${\hat{\gamma }}_{p}^{g}$ remained in the population. From (19), it could be seen that the smaller value of the MLF meant the better fitness. The selection stage was described by the following equations:

(20) $$\begin{array}{ccc} \lambda ({\hat{\eta }}_{p}^{g+1}) & = & \frac{1}{k_{\max}}\sum_{k=1}^{k_{\max}}{\left[M_{R}\left(k\right)-\tau^{T}\left(k\right){\hat{\eta }}_{p}^{g+1}\right]}^{2}, \end{array}$$

(21) $$\begin{array}{ccc} \lambda ({\hat{\gamma }}_{p}^{g}) & = & \frac{1}{k_{\max}}\sum_{k=1}^{k_{\max}}{\left[M_{R}\left(k\right)-\tau^{T}\left(k\right){\hat{\gamma }}_{p}^{g}\right]}^{2}, \end{array}$$

(22) $$\begin{array}{ccc} {\hat{\gamma }}_{p}^{g+1} & = & \left\{\begin{array}{cc} {\hat{\eta }}_{p}^{g+1}, & \mathrm{if}\;\lambda \left({\hat{\eta }}_{p}^{g+1}\right)<\lambda \left({\hat{\gamma }}_{p}^{g}\right)\\ {\hat{\gamma }}_{p}^{g}, & \mathrm{if}\;\lambda \left({\hat{\eta }}_{p}^{g+1}\right)⩾\lambda \left({\hat{\gamma }}_{p}^{g}\right) \end{array}\right.. \end{array}$$

According to (22), if the MLF of the crossover vector ${\hat{\eta }}_{p}^{g+1}$ is smaller, the individual ${\hat{\gamma }}_{p}^{g+1}$ at the generation g+1 will be taken the place of the crossover vector ${\hat{\eta }}_{p}^{g+1}$; otherwise, the individual ${\hat{\gamma }}_{p}^{g}$ will remain in the population until the generation g+1.

Afterwards, we could find the best individual ${\hat{\gamma }}_{best}^{g+1}$ at the generation g+1 in the population ${\hat{\Gamma }}^{g+1}={\left[{\hat{\gamma }}_{1}^{g+1},{\hat{\gamma }}_{2}^{g+1},\cdots ,{\hat{\gamma }}_{P}^{g+1}\right]}^{T}$ through the following equation:

(23) $$\begin{array}{ccc} {\hat{\gamma }}_{best}^{g+1} & = & \underset{{\hat{\gamma }}_{p}^{g+1}}{\arg\min}\lambda \left({\hat{\gamma }}_{p}^{g+1}\right)\\ & = & \underset{{\hat{\gamma }}_{p}^{g+1}}{\arg\min}\frac{1}{k_{\max}}\sum_{k=1}^{k_{\max}}{\left[M_{R}\left(k\right)-\tau^{T}\left(k\right){\hat{\gamma }}_{p}^{g+1}\right]}^{2},\;p=1,2,\cdots ,P. \end{array}$$

When $g<g_{\max}-1$, let *g* increase by 1 and repeat the mutation stage, the crossover stage, and the selection stage to update the individual ${\hat{\gamma }}_{p}^{g+1}$ in the population ${\hat{\Gamma }}^{g+1}$. When $g=g_{\max}-1$, the best individual ${\hat{\gamma }}_{best}^{g_{\max}}$ is the final estimate of the parameter vector γ.

Combining (4)–(12) and (20)–(23), we have the MLP-IE algorithm to estimate the parameter vector γ of the model in (3):

(24) $$\begin{array}{ccc} {\hat{\Gamma }}^{0} & = & {\left[{\hat{\gamma }}_{1}^{0},{\hat{\gamma }}_{2}^{0},\cdots ,{\hat{\gamma }}_{P}^{0}\right]}^{T}, \end{array}$$

(25) $$\begin{array}{ccc} {\hat{\gamma }}_{p}^{0} & = & {\left[{\hat{\gamma }}_{p,1}^{0},{\hat{\gamma }}_{p,2}^{0},\cdots ,{\hat{\gamma }}_{p,Q}^{0}\right]}^{T},\;p=1,2,\cdots ,P, \end{array}$$

(26) $$\begin{array}{ccc} {\hat{\gamma }}_{p,q}^{0} & = & \mathrm{rand}(0,1),\;q=1,2,\cdots ,Q, \end{array}$$

(27) $$\begin{array}{ccc} {\hat{\zeta }}_{p}^{g+1} & = & {\hat{\gamma }}_{\kappa_{1}}^{g}+F\cdot \left({\hat{\gamma }}_{\kappa_{2}}^{g}-{\hat{\gamma }}_{\kappa_{3}}^{g}\right),\;\kappa_{1},\kappa_{2},\kappa_{3}\in \left[1,P\right], \end{array}$$

(28) $$\begin{array}{ccc} {\hat{\zeta }}_{p}^{g} & = & {\left[{\hat{\zeta }}_{p,1}^{g},{\hat{\zeta }}_{p,2}^{g},\cdots ,{\hat{\zeta }}_{p,Q}^{g}\right]}^{T}, \end{array}$$

(29) $$\begin{array}{ccc} {\hat{\eta }}_{p,q}^{g+1} & = & \left\{\begin{array}{cc} {\hat{\zeta }}_{p,q}^{g+1}, & \mathrm{if}\;\mathrm{rand}\left(0,1\right)<C_{R}\;\mathrm{or}\;q=q_{\mathrm{rand}}\\ {\hat{\gamma }}_{p,q}^{g}, & \mathrm{if}\;\mathrm{rand}\left(0,1\right)⩾C_{R}\;\mathrm{and}\;q\neq q_{\mathrm{rand}} \end{array}\right., \end{array}$$

(30) $$\begin{array}{ccc} {\hat{\eta }}_{p}^{g} & = & {\left[{\hat{\eta }}_{p,1}^{g},{\hat{\eta }}_{p,2}^{g},\cdots ,{\hat{\eta }}_{p,Q}^{g}\right]}^{T}, \end{array}$$

(31) $$\begin{array}{ccc} \lambda ({\hat{\eta }}_{p}^{g+1}) & = & \frac{1}{k_{\max}}\sum_{k=1}^{k_{\max}}{\left[M_{R}\left(k\right)-\tau^{T}\left(k\right){\hat{\eta }}_{p}^{g+1}\right]}^{2}, \end{array}$$

(32) $$\begin{array}{ccc} \lambda ({\hat{\gamma }}_{p}^{g}) & = & \frac{1}{k_{\max}}\sum_{k=1}^{k_{\max}}{\left[M_{R}\left(k\right)-\tau^{T}\left(k\right){\hat{\gamma }}_{p}^{g}\right]}^{2}, \end{array}$$

(33) $$\begin{array}{ccc} {\hat{\gamma }}_{p}^{g+1} & = & \left\{\begin{array}{cc} {\hat{\eta }}_{p}^{g+1}, & \mathrm{if}\;\lambda \left({\hat{\eta }}_{p}^{g+1}\right)<\lambda \left({\hat{\gamma }}_{p}^{g}\right)\\ {\hat{\gamma }}_{p}^{g}, & \mathrm{if}\;\lambda \left({\hat{\eta }}_{p}^{g+1}\right)⩾\lambda \left({\hat{\gamma }}_{p}^{g}\right) \end{array}\right., \end{array}$$

(34) $$\begin{array}{ccc} {\hat{\Gamma }}^{g} & = & {\left[{\hat{\gamma }}_{1}^{g},{\hat{\gamma }}_{2}^{g},\cdots ,{\hat{\gamma }}_{P}^{g}\right]}^{T}, \end{array}$$

(35) $$\begin{array}{ccc} {\hat{\gamma }}_{p}^{g} & = & {\left[{\hat{\gamma }}_{p,1}^{g},{\hat{\gamma }}_{p,2}^{g},\cdots ,{\hat{\gamma }}_{p,Q}^{g}\right]}^{T}, \end{array}$$

(36) $$\begin{array}{ccc} {\hat{\gamma }}_{best}^{g+1} & = & \underset{{\hat{\gamma }}_{p}^{g+1}}{\arg\min}\frac{1}{k_{\max}}\sum_{k=1}^{k_{\max}}{\left[M_{R}\left(k\right)-\tau^{T}\left(k\right){\hat{\gamma }}_{p}^{g+1}\right]}^{2}. \end{array}$$

**Figure 3:**
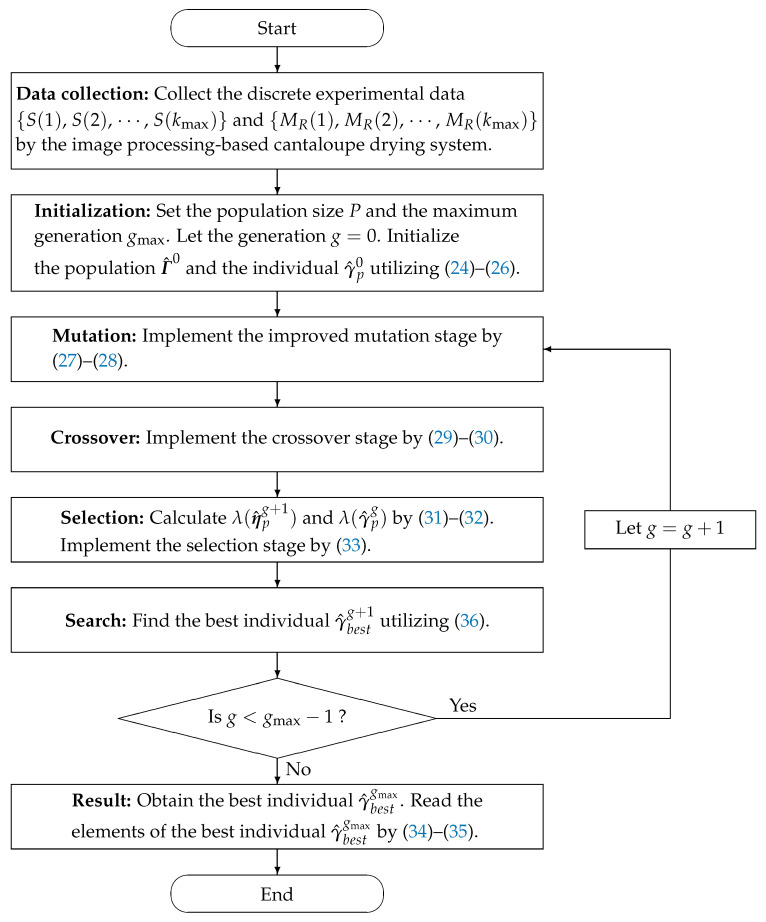
The flowchart of the MLP-IE algorithm.

The flowchart of utilizing the MLP-IE algorithm in (24)–(36) to estimate the parameter vector γ of the model in (3) is displayed in Figure 3.

The pseudo codes of the MLP-IE algorithm in (24)–(36) are displayed in Algorithm 1.

**Algorithm 1:** The pseudo codes of the MLP-IE algorithm

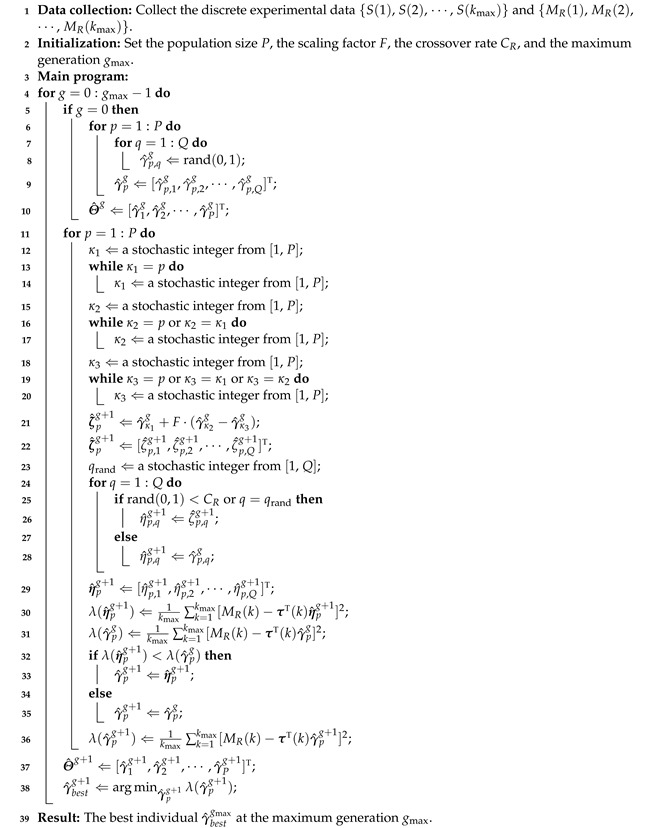



## 4. MLP-I-IE Algorithm

To enhance the model fitting ability of the MLP-IE algorithm, this section proposed the maximum likelihood principle-based improved iterative evolution (MLP-I-IE) algorithm by utilizing the improved mutation strategy, the improved scaling factor and the improved crossover rate.

### 4.1. Improved Mutation Strategy

From the mutation stage in (10), it could be seen that the three individuals ${\hat{\gamma }}_{\kappa_{1}}^{g}$, ${\hat{\gamma }}_{\kappa_{2}}^{g}$, and ${\hat{\gamma }}_{\kappa_{3}}^{g}$ were stochastically selected from the population ${\hat{\Gamma }}^{g}$. In order to enhance the performance of the MLP-IE algorithm, we proposed an improved mutation strategy. In the improved mutation strategy, the three stochastically selected individuals ${\hat{\gamma }}_{\kappa_{1}}^{g}$, ${\hat{\gamma }}_{\kappa_{2}}^{g}$, and ${\hat{\gamma }}_{\kappa_{3}}^{g}$ were sorted by their MLFs, where the individual with the best MLF is recorded as ${\hat{\gamma }}_{\kappa_{best}}^{g}$, the individual with the suboptimal MLF is recorded as ${\hat{\gamma }}_{\kappa_{sub}}^{g}$ and the individual with the worst MLF is recorded as ${\hat{\gamma }}_{\kappa_{worst}}^{g}$. The mutant vector ${\hat{\zeta }}_{p}^{g+1}$ was obtained by adding the vectorial difference between the individual with the suboptimal MLF ${\hat{\gamma }}_{\kappa_{sub}}^{g}$ and the individual with the worst MLF ${\hat{\gamma }}_{\kappa_{worst}}^{g}$ to the individual with the best MLF ${\hat{\gamma }}_{\kappa_{best}}^{g}$:

(37) $${\hat{\zeta }}_{p}^{g+1}={\hat{\gamma }}_{\kappa_{best}}^{g}+F\cdot \left({\hat{\gamma }}_{\kappa_{sub}}^{g}-{\hat{\gamma }}_{\kappa_{worst}}^{g}\right),\;\kappa_{best},\kappa_{sub},\kappa_{worst}\in \left\{\kappa_{1},\kappa_{2},\kappa_{3}\right\},\;\kappa_{1},\kappa_{2},\kappa_{3}\in \left[1,P\right].$$

It could be seen from (37) that the improved mutation strategy was no longer random search, but the base vector ${\hat{\gamma }}_{\kappa_{best}}^{g}$ searched in the direction of ${\hat{\gamma }}_{\kappa_{sub}}^{g}-{\hat{\gamma }}_{\kappa_{worst}}^{g}$. It was worth noting that $\kappa_{1}$, $\kappa_{2}$, and $\kappa_{3}$ stochastically selected from the set {1, 2, ⋯, P} were still not equal to each other and not equal to the index *p* and $\kappa_{best}$, $\kappa_{sub}$, and $\kappa_{worst}$ were chosen from $\left\{\kappa_{1},\kappa_{2},\kappa_{3}\right\}$.

### 4.2. Improved Scaling Factor

Additionally, it could be observed from (10) that the scaling factor *F* controlled the magnitude of the vectorial difference ${\hat{\gamma }}_{\kappa_{2}}^{g}-{\hat{\gamma }}_{\kappa_{3}}^{g}$. Thus we proposed an improved scaling factor $F^{g+1}$ that was changed with the generation g+1 to improve the performance of the MLP-IE algorithm:

(38) $$F^{g+1}=f_{1}\cdot e^{-{\left(f_{2}\cdot \left(g+1\right)/g_{\max}\right)}^{f_{3}}},$$

where $f_{1}$, $f_{2}$, and $f_{3}$ are positive constants. From the improved mutation strategy in (37) and the improved scaling factor $F^{g+1}$ in (38), we could see that for the small generation g+1, $F^{g+1}$ was large enough to extend the range of the MLP-I-IE algorithm to search the optimal solution and as the generation g+1 increased, $F^{g+1}$ gradually decreased to accelerate the convergence speed. Compared with the scaling factor *F* in the MLP-IE algorithm, the improved scaling factor $F^{g+1}$ took into account both the global search ability and the convergence speed.

### 4.3. Improved Crossover Rate

From the crossover stage in (12), we knew that the crossover rate $C_{R}$ determined that the element in the crossover vector ${\hat{\eta }}_{p}^{g+1}$ preserved the element in the mutant vector ${\hat{\zeta }}_{p}^{g+1}$ or the element in the individual ${\hat{\gamma }}_{p}^{g}$. Therefore, we proposed an improved crossover rate $C_{R}^{g+1}$ to enhance the performance of the MLP-IE algorithm:

(39) $$\begin{array}{ccc} C_{R}^{g+1} & = & \left\{\begin{array}{cc} \left[1+\sin\left(g+1\right)\right]/c_{1}+c_{2}, & \mathrm{if}\;g+1=1\;\mathrm{or}\;g+1=2l\\ \left[1+\sin\left(g\right)\right]/c_{1}+c_{2}, & \mathrm{if}\;g+1=2l+1 \end{array}\right., \end{array}$$

where $c_{1}$ and $c_{2}$ are positive constants. The improved crossover rate $C_{R}^{g+1}$ in (39) changed versus the generation g+1 and updated once as the generation g+1 changed twice. As the improved crossover rate $C_{R}^{g+1}$ constantly changed, ${\hat{\eta }}_{p,q}^{g+1}$ preserved ${\hat{\zeta }}_{p,q}^{g+1}$ or ${\hat{\gamma }}_{p,q}^{g}$ stochastically, which helped the MLP-I-IE algorithm jump from the local optimal solution. From the crossover stage in (11) and the improved crossover rate $C_{R}^{g+1}$ in (39), we could see that for the small crossover rate $C_{R}^{g+1}$, the probability of keeping ${\hat{\zeta }}_{p,q}^{g+1}$ in ${\hat{\eta }}_{p,q}^{g+1}$ was large while for the large crossover rate $C_{R}^{g+1}$, the probability of keeping ${\hat{\gamma }}_{p,q}^{g}$ in ${\hat{\eta }}_{p,q}^{g+1}$ was large.

Combining the MLP-IE algorithm in (4)–(11) and (20)–(23), the improved mutation strategy in (37), the improved scaling factor in (38) and the improved crossover rate in (39), we have the MLP-I-IE algorithm to estimate the parameter vector γ of the model in (3):

(40) $$\begin{array}{ccc} {\hat{\Gamma }}^{0} & = & {\left[{\hat{\gamma }}_{1}^{0},{\hat{\gamma }}_{2}^{0},\cdots ,{\hat{\gamma }}_{P}^{0}\right]}^{T}, \end{array}$$

(41) $$\begin{array}{ccc} {\hat{\gamma }}_{p}^{0} & = & {\left[{\hat{\gamma }}_{p,1}^{0},{\hat{\gamma }}_{p,2}^{0},\cdots ,{\hat{\gamma }}_{p,Q}^{0}\right]}^{T},\;p=1,2,\cdots ,P, \end{array}$$

(42) $$\begin{array}{ccc} {\hat{\gamma }}_{p,q}^{0} & = & \mathrm{rand}(0,1),\;q=1,2,\cdots ,Q, \end{array}$$

(43) $$\begin{array}{ccc} F^{g+1} & = & f_{1}\cdot e^{-{\left(f_{2}\cdot \left(g+1\right)/g_{\max}\right)}^{f_{3}}},\\ {\hat{\zeta }}_{p}^{g+1} & = & {\hat{\gamma }}_{\kappa_{best}}^{g}+F^{g+1}\cdot \left({\hat{\gamma }}_{\kappa_{sub}}^{g}-{\hat{\gamma }}_{\kappa_{worst}}^{g}\right), \end{array}$$

(44) $$\begin{array}{ccc} & & \kappa_{best},\kappa_{sub},\kappa_{worst}\in \left\{\kappa_{1},\kappa_{2},\kappa_{3}\right\},\;\kappa_{1},\kappa_{2},\kappa_{3}\in \left[1,P\right], \end{array}$$

(45) $$\begin{array}{ccc} {\hat{\zeta }}_{p}^{g+1} & = & {\left[{\hat{\zeta }}_{p,1}^{g+1},{\hat{\zeta }}_{p,2}^{g+1},\cdots ,{\hat{\zeta }}_{p,Q}^{g+1}\right]}^{T}, \end{array}$$

(46) $$\begin{array}{ccc} C_{R}^{g+1} & = & \left\{\begin{array}{cc} \left[1+\sin\left(g+1\right)\right]/c_{1}+c_{2}, & \mathrm{if}\;g+1=1\;\mathrm{or}\;g+1=2l\\ \left[1+\sin\left(g\right)\right]/c_{1}+c_{2}, & \mathrm{if}\;g+1=2l+1 \end{array}\right., \end{array}$$

(47) $$\begin{array}{ccc} {\hat{\eta }}_{p,q}^{g+1} & = & \left\{\begin{array}{cc} {\hat{\zeta }}_{p,q}^{g+1}, & \mathrm{if}\;\mathrm{rand}\left(0,1\right)<C_{R}^{g+1}\;\mathrm{or}\;q=q_{\mathrm{rand}}\\ {\hat{\gamma }}_{p,q}^{g}, & \mathrm{if}\;\mathrm{rand}\left(0,1\right)⩾C_{R}^{g+1}\;\mathrm{and}\;q\neq q_{\mathrm{rand}} \end{array}\right., \end{array}$$

(48) $$\begin{array}{ccc} {\hat{\eta }}_{p}^{g+1} & = & {\left[{\hat{\eta }}_{p,1}^{g+1},{\hat{\eta }}_{p,2}^{g+1},\cdots ,{\hat{\eta }}_{p,Q}^{g}\right]}^{T}, \end{array}$$

(49) $$\begin{array}{ccc} \lambda ({\hat{\eta }}_{p}^{g+1}) & = & \frac{1}{k_{\max}}\sum_{k=1}^{k_{\max}}{\left[M_{R}\left(k\right)-\tau^{T}\left(k\right){\hat{\eta }}_{p}^{g+1}\right]}^{2}, \end{array}$$

(50) $$\begin{array}{ccc} \lambda ({\hat{\gamma }}_{p}^{g}) & = & \frac{1}{k_{\max}}\sum_{k=1}^{k_{\max}}{\left[M_{R}\left(k\right)-\tau^{T}\left(k\right){\hat{\gamma }}_{p}^{g}\right]}^{2}, \end{array}$$

(51) $$\begin{array}{ccc} {\hat{\gamma }}_{p}^{g+1} & = & \left\{\begin{array}{c} {\hat{\eta }}_{p}^{g+1},\mathrm{if}\;\lambda \left({\hat{\eta }}_{p}^{g+1}\right)<\lambda \left({\hat{\gamma }}_{p}^{g}\right)\\ {\hat{\gamma }}_{p}^{g},\mathrm{if}\;\lambda \left({\hat{\eta }}_{p}^{g+1}\right)⩾\lambda \left({\hat{\gamma }}_{p}^{g}\right) \end{array}\right., \end{array}$$

(52) $$\begin{array}{ccc} {\hat{\gamma }}_{p}^{g+1} & = & {\left[{\hat{\gamma }}_{p,1}^{g+1},{\hat{\gamma }}_{p,2}^{g+1},\cdots ,{\hat{\gamma }}_{p,Q}^{g+1}\right]}^{T}, \end{array}$$

(53) $$\begin{array}{ccc} {\hat{\Gamma }}^{g+1} & = & {\left[{\hat{\gamma }}_{1}^{g+1},{\hat{\gamma }}_{2}^{g+1},\cdots ,{\hat{\gamma }}_{P}^{g+1}\right]}^{T}, \end{array}$$

(54) $$\begin{array}{ccc} {\hat{\gamma }}_{best}^{g+1} & = & \underset{{\hat{\gamma }}_{p}^{g+1}}{\arg\min}\frac{1}{k_{\max}}\sum_{k=1}^{k_{\max}}{\left[M_{R}\left(k\right)-\tau^{T}\left(k\right){\hat{\gamma }}_{p}^{g+1}\right]}^{2}. \end{array}$$

**Figure 4:**
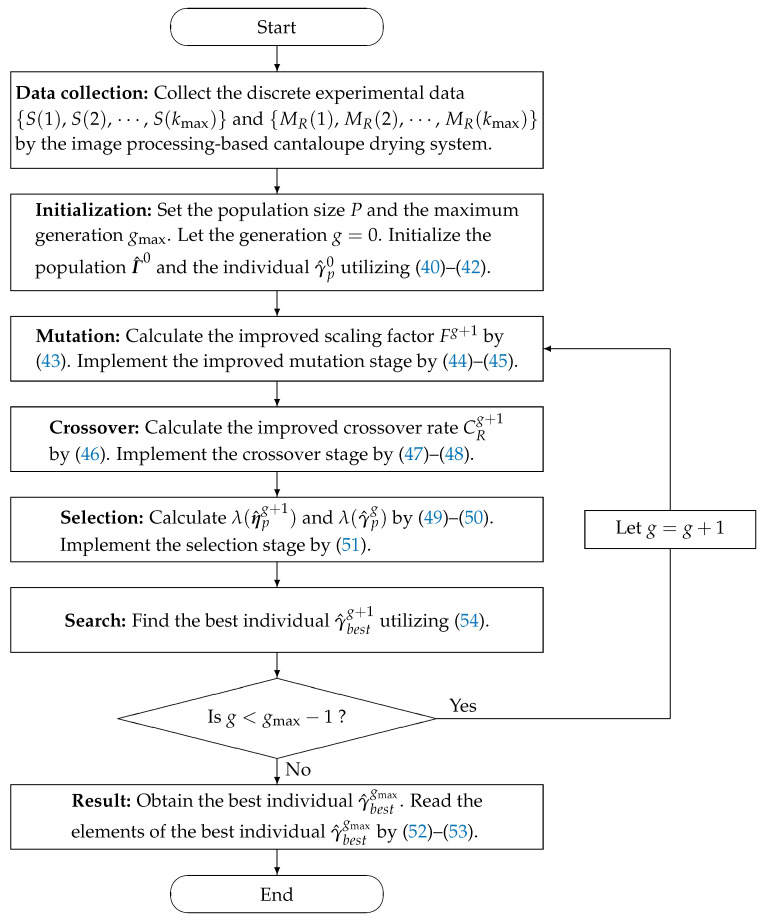
The flowchart of the MLP-I-IE algorithm.

The flowchart of utilizing the MLP-I-IE algorithm in (40)–(54) to estimate the parameter vector γ of the model in (3) is displayed in Figure 4.

The pseudo codes of the MLP-I-IE algorithm in (40)–(54) are displayed in Algorithm 2.

**Algorithm 2:** The pseudo codes of the MLP-I-IE algorithm

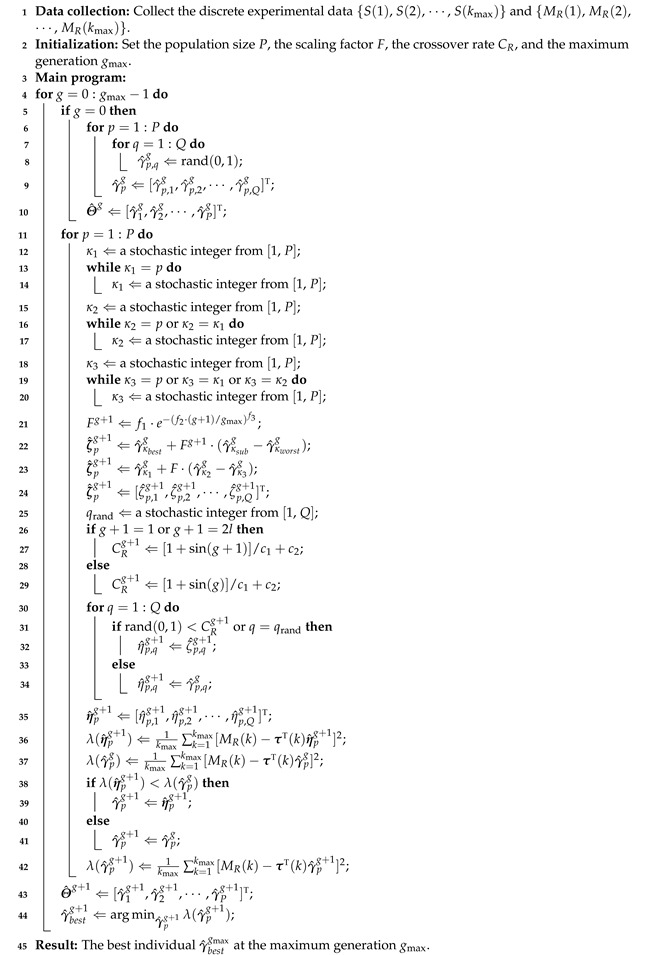



## 5. Estimation of the Moisture Ratio Model

### 5.1. Model Estimation

In model estimation, a batch of discrete experimental data {S(k), $M_{R}\left(k\right):$k=1, 2, ⋯, $k_{\max}\}$ during the drying process of cantaloupe slices sampled by the developed image processing-based microwave drying system were used. The orders n=1, n=2, and n=3 were set for the model in (3), and the MLP-IE algorithm and MLP-I-IE algorithm (the population size P=30 and the maximum generation $g_{\max}=20$) were exploited to produce the parameter estimates. To evaluate the model fitting ability of the MLP-IE algorithm and MLP-I-IE algorithm and choose the optimal order, the fitting coefficient was defined as

$$R^{2}=1-\frac{\sum_{k=1}^{k_{\max}}{\left[{\hat{M}}_{R,est}\left(k\right)-M_{R}\left(k\right)\right]}^{2}}{\sum_{k=1}^{k_{\max}}{\left[M_{R}\left(k\right)-\bar{M_{R}}\right]}^{2}}=1-\frac{\sum_{k=1}^{k_{\max}}{\left[\tau^{T}\left(k\right)\hat{\gamma }-M_{R}\left(k\right)\right]}^{2}}{\sum_{k=1}^{k_{\max}}{\left[M_{R}\left(k\right)-\bar{M_{R}}\right]}^{2}},$$

where ${\hat{M}}_{R,est}\left(k\right)$ is the estimated moisture ratio, $M_{R}\left(k\right)$ is the experimental moisture ratio and $\bar{M_{R}}$ is the average value of the experimental moisture ratios. Nevertheless, the model orders also affect the value of $R^{2}$. If the model orders are different, $R^{2}$ cannot be used to evaluate the model fitting ability. The adjusted fitting coefficient $adjR^{2}$ was defined as

$$adjR^{2}=1-\left(1-R^{2}\right)\frac{\left(k_{\max}-1\right)}{k_{\max}-\left(n+1\right)},$$

which considers both the number of discrete experimental data $k_{\max}$ and the number of parameters to be estimated n+1.

Moreover, the root-mean-square error (RMSE) was also used to evaluate the model fitting ability and it was defined as

$$\mathrm{RMSE}=\sqrt{\frac{1}{k_{\max}}\sum_{k=1}^{k_{\max}}{\left[{\hat{M}}_{R,est}\left(k\right)-M_{R}\left(k\right)\right]}^{2}}=\sqrt{\frac{1}{k_{\max}}\sum_{k=1}^{k_{\max}}{\left[\tau^{T}\left(k\right)\hat{\gamma }-M_{R}\left(k\right)\right]}^{2}}.$$

**Figure 5:**
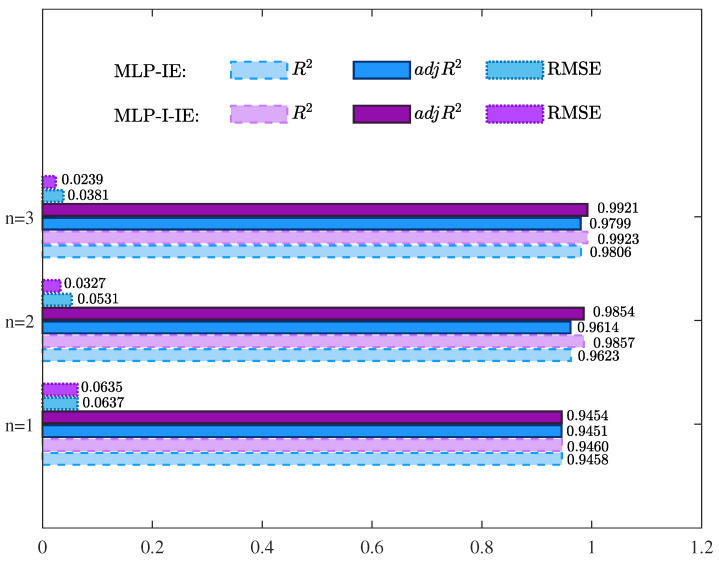
The values of $R^{2}$, $adjR^{2}$, and RMSEs of the estimated models given by different algorithms for different orders *n*.

**Figure 6:**
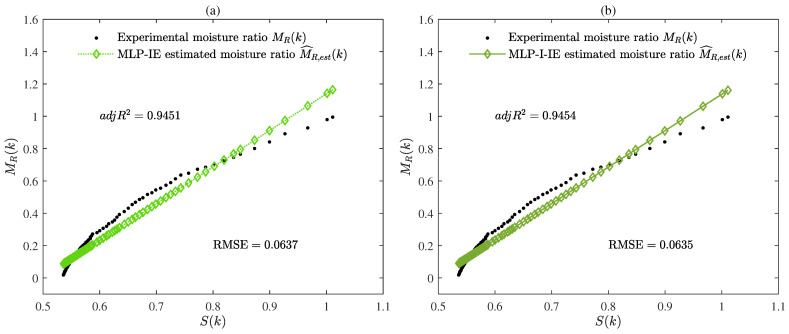
(**a**) The estimated moisture ratio ${\hat{M}}_{R,est}\left(k\right)$ given by the MLP-IE algorithm versus the shrinkage S(k) for n=1; (**b**) the estimated moisture ratio ${\hat{M}}_{R,est}\left(k\right)$ given by the MLP-I-IE algorithm versus the shrinkage S(k) for n=1.

**Figure 7:**
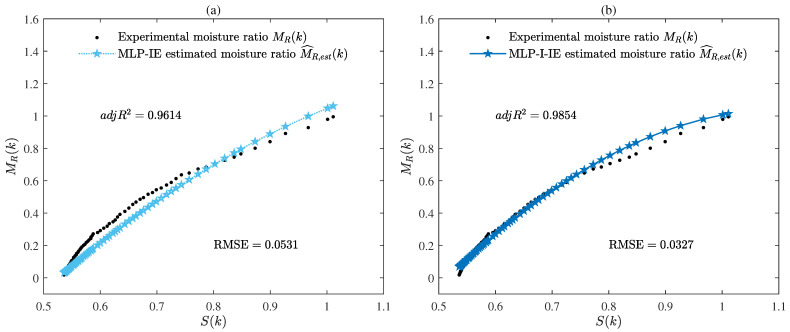
(**a**) The estimated moisture ratio ${\hat{M}}_{R,est}\left(k\right)$ given by the MLP-IE algorithm versus the shrinkage S(k) for n=2; (**b**) the estimated moisture ratio ${\hat{M}}_{R,est}\left(k\right)$ given by the MLP-I-IE algorithm versus the shrinkage S(k) for n=2.

**Figure 8:**
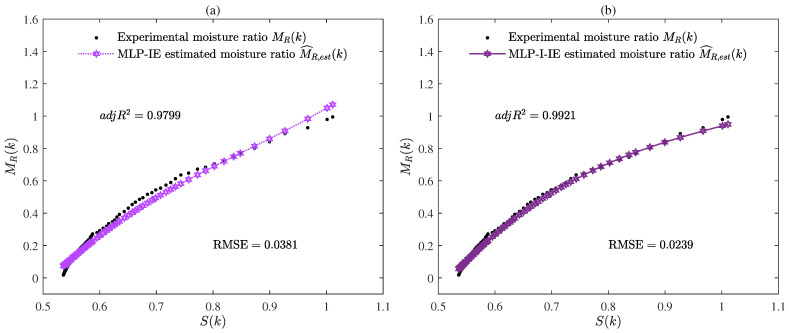
(**a**) The estimated moisture ratio ${\hat{M}}_{R,est}\left(k\right)$ given by the MLP-IE algorithm versus the shrinkage S(k) for n=3; (**b**) the estimated moisture ratio ${\hat{M}}_{R,est}\left(k\right)$ given by the MLP-I-IE algorithm versus the shrinkage S(k) for n=3.

The parameter estimates given by the MLP-IE algorithm and MLP-I-IE algorithm for n=1, n=2, and n=3 are summarized in Table 1. Besides, the values of $R^{2}$, $adjR^{2}$, and RMSE given by the MLP-IE algorithm and MLP-I-IE algorithm for n=1, n=2, and n=3 are also shown in Table 1 and Figure 5. The curves of the estimated moisture ratio ${\hat{M}}_{R,est}\left(k\right)$ and the experimental moisture ratio $M_{R}\left(k\right)$ versus the shrinkage S(k) for n=1, n=2, and n=3 are depicted in Figure 6, Figure 7 and Figure 8.

**Figure 9:**
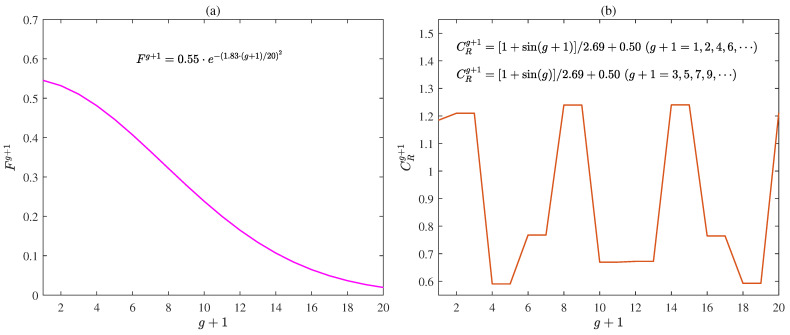
(**a**) The improved scaling factor $F^{g+1}$ of the MLP-I-IE algorithm for n=3; (**b**) the improved crossover rate $C_{R}^{g+1}$ of the MLP-I-IE algorithm for n=3.

The improved scaling factor $F^{g+1}$ and the improved crossover rate $C_{R}^{g+1}$ of the MLP-I-IE algorithm versus the generation g+1 for n=3 are illustrated in Figure 9.

### 5.2. Model Validation

In model validation, another batch of discrete experimental data {S(k), $M_{R}\left(k\right):\;$k=1, 2, ⋯, $k_{\max}\}$ during the drying process of cantaloupe slices sampled by the developed image processing-based microwave drying system were used. The parameter estimates $\hat{\gamma }$ given by the MLP-IE algorithm and MLP-I-IE algorithm for n=3 were used to compute the predicted moisture ratio ${\hat{M}}_{R,pre}\left(k\right)$. The fitting coefficient $R^{2}$ of the predicted model was computed by

$$R^{2}=1-\frac{\sum_{k=1}^{k_{\max}}{\left[{\hat{M}}_{R,pre}\left(k\right)-M_{R}\left(k\right)\right]}^{2}}{\sum_{k=1}^{k_{\max}}{\left[M_{R}\left(k\right)-\bar{M_{R}}\right]}^{2}}=1-\frac{\sum_{k=1}^{k_{\max}}{\left[\tau^{T}\left(k\right)\hat{\gamma }-M_{R}\left(k\right)\right]}^{2}}{\sum_{k=1}^{k_{\max}}{\left[M_{R}\left(k\right)-\bar{M_{R}}\right]}^{2}},$$

the adjusted fitting coefficient $adjR^{2}$ of the predicted model was computed by

$$adjR^{2}=1-\left(1-R^{2}\right)\frac{\left(k_{\max}-1\right)}{k_{\max}-\left(n+1\right)},$$

and the RMSE of the predicted model was computed by

$$\;\mathrm{RMSE}=\sqrt{\frac{1}{k_{\max}}\sum_{k=1}^{k_{\max}}{\left[{\hat{M}}_{R,pre}\left(k\right)-M_{R}\left(k\right)\right]}^{2}}=\sqrt{\frac{1}{k_{\max}}\sum_{k=1}^{k_{\max}}{\left[\tau^{T}\left(k\right)\hat{\gamma }-M_{R}\left(k\right)\right]}^{2}}.$$

**Figure 10:**
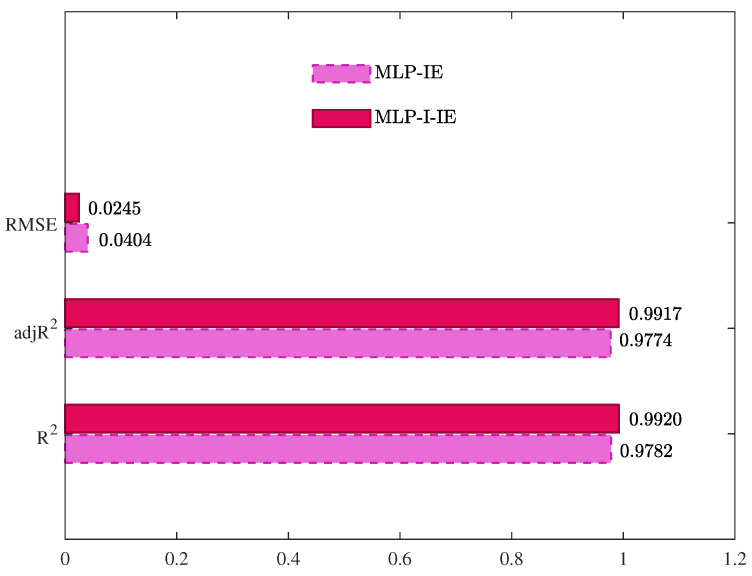
The values of $R^{2}$, $adjR^{2}$, and RMSEs of the estimated models given by different algorithms for different orders *n*.

**Figure 11:**
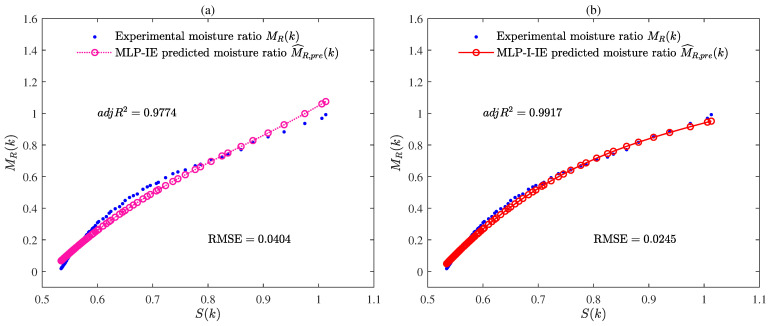
(**a**) The predicted moisture ratio ${\hat{M}}_{R,pre}\left(k\right)$ given by the MLP-IE algorithm versus the shrinkage S(k); (**b**) the predicted moisture ratio ${\hat{M}}_{R,pre}\left(k\right)$ given by the MLP-I-IE algorithm versus the shrinkage S(k).

**Figure 12:**
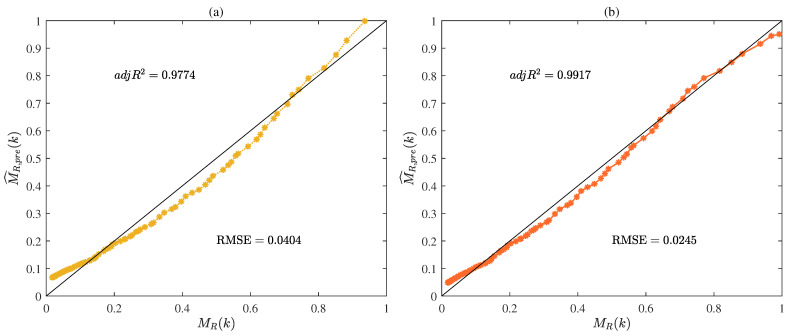
(**a**) The predicted moisture ratio ${\hat{M}}_{R,pre}\left(k\right)$ given by the MLP-IE algorithm versus the experimental moisture ratio $M_{R}\left(k\right)$; (**b**) the predicted moisture ratio ${\hat{M}}_{R,pre}\left(k\right)$ given by the MLP-I-IE algorithm versus versus the experimental moisture ratio $M_{R}\left(k\right)$.

The values of $R^{2}$, $adjR^{2}$, and RMSE given by the MLP-IE algorithm and the MLP-I-IE algorithm for the predicted model are shown in Table 2 and Figure 10. The curves of the predicted moisture ratio ${\hat{M}}_{R,pre}\left(k\right)$ versus the shrinkage S(k) are illustrated in Figure 11. The comparisons of the predicted moisture ratio ${\hat{M}}_{R,pre}\left(k\right)$ and the experimental moisture ratio $M_{R}\left(k\right)$ are illustrated in Figure 12.

The following conclusions were deduced in accordance with the results in Table 1 and Table 2 and Figure 5, Figure 6, Figure 7, Figure 8, Figure 9, Figure 10, Figure 11 and Figure 12.

It could be deduced from Table 1 and Figure 5, Figure 6, Figure 7, Figure 8 and Figure 9 that both the MLP-IE algorithm and MLP-I-IE algorithm were effective for model fitting because the estimated moisture ratios ${\hat{M}}_{R,est}\left(k\right)$ given by the two algorithms were close to the experimental moisture ratio $M_{R}\left(k\right)$. For the same order, the adjusted fitting coefficient $adjR^{2}$ of the estimated model given by the MLP-I-IE algorithm was larger than that given by the MLP-IE algorithm, and the RMSE of the estimated model given by the MLP-I-IE algorithm was smaller than that given by the MLP-IE algorithm, which meant the MLP-I-IE algorithm performed better than the MLP-IE algorithm in model fitting. Meanwhile, n=3 was selected as the optimal order for the reason that the adjusted fitting coefficients $adjR^{2}$ of the estimated models given by the MLP-IE algorithm and the MLP-I-IE algorithm for n=3 were larger than $adjR^{2}$ for n=1 and n=2, and the RMSEs of the estimated models given by the MLP-IE algorithm and the MLP-I-IE algorithm for n=3 were smaller than RMSEs for n=1 and n=2.

It could be seen from Table 2 and Figure 10, Figure 11 and Figure 12 that the predicted model given by the MLP-I-IE algorithm had the larger $adjR^{2}$ and the smaller RMSE than that given by the MLP-IE algorithm, indicating that the predicted model given by the MLP-I-IE algorithm was better than that given by the MLP-IE algorithm. In other words, the predicted moisture ratio ${\hat{M}}_{R,pre}\left(k\right)$ given by the MLP-I-IE algorithm was closer to the MLP-IE algorithm. Therefore, the proposed MLP-I-IE algorithm had more excellent performance in estimation and prediction and could be utilized to predict the moisture ratio model of the cantaloupe microwave drying process.

## 6. Conclusions

In this paper, an image processing-based cantaloupe drying system was designed to generate the experimental data and the mathematical model of the moisture ratio with regard to the shrinkage during the drying process of cantaloupe slices was built according to Weierstrass approximation theorem. By deriving the maximum likelihood fitness, the MLP-IE algorithm was developed to estimate the moisture ratio model. Afterwards, by designing the improved mutation strategy, the improved scaling factor, and the improved crossover rate, we proposed the MLP-I-IE algorithm to strengthen the model fitting ability of the MLP-IE algorithm. Finally, the results revealed that the two presented algorithms were effective for model fitting, and that the MLP-I-IE algorithm was able to estimate a more accurate model than the MLP-IE algorithm. In our future work, we will study adjusted algorithms with better prediction effects and develop different control strategies to improve the quality of drying products during food microwave-drying processes.

## Figures and Tables

**Table 1:** The values of $R^{2}$, $adjR^{2}$, and RMSE of the estimated models given by different algorithms.

*n*	Algorithms	${\hat{\gamma }}_{0,\mathrm{best}}^{g_{\max}}$	${\hat{\gamma }}_{1,\mathrm{best}}^{g_{\max}}$	${\hat{\gamma }}_{2,\mathrm{best}}^{g_{\max}}$	${\hat{\gamma }}_{3,\mathrm{best}}^{g_{\max}}$	$R^{2}$	${\mathrm{adjR}}^{2}$	RMSE
ine 1	MLP-IE	−1.1281	2.2675	–	–	0.9458	0.9451	0.0637
	MLP-I-IE	−1.1187	2.2551	–	–	0.9460	0.9454	0.0635
ine 2	MLP-IE	−2.0551	4.8087	−1.7074	–	0.9623	0.9614	0.0531
	MLP-I-IE	−2.5458	6.4070	−2.8547	–	0.9857	0.9854	0.0327
ine 3	MLP-IE	−3.7212	12.3373	−12.4019	4.8339	0.9806	0.9799	0.0381
	MLP-I-IE	−4.0180	12.5609	−11.1670	3.5632	0.9923	0.9921	0.0239

**Table 2:** The values of $R^{2}$, $adjR^{2}$, and RMSE of the predicted models given by different algorithms.

Algorithms	$R^{2}$	${\mathrm{adjR}}^{2}$	RMSE
MLP-IE	0.9782	0.9774	0.0404
MLP-I-IE	0.9920	0.9917	0.0245

## Data Availability

The data in this research are available upon request from the corresponding author.
